# Ten simple rules for running a virtual program to introduce computational biology at the high school level

**DOI:** 10.1371/journal.pcbi.1013830

**Published:** 2025-12-29

**Authors:** Hanako Osuga, Matthew C. Chan, Katherine Brower, Liza J. Ray, Jeanne T. Chowning

**Affiliations:** 1 Science Education, Fred Hutchinson Cancer Center, Seattle, Washington, United States of America; 2 Basic Sciences Division, Fred Hutchinson Cancer Center, Seattle, Washington, United States of America; 3 Department of Genome Sciences, University of Washington, Seattle, Washington, United States of America; Carnegie Mellon University, UNITED STATES OF AMERICA

## Introduction

**Fig 1:**
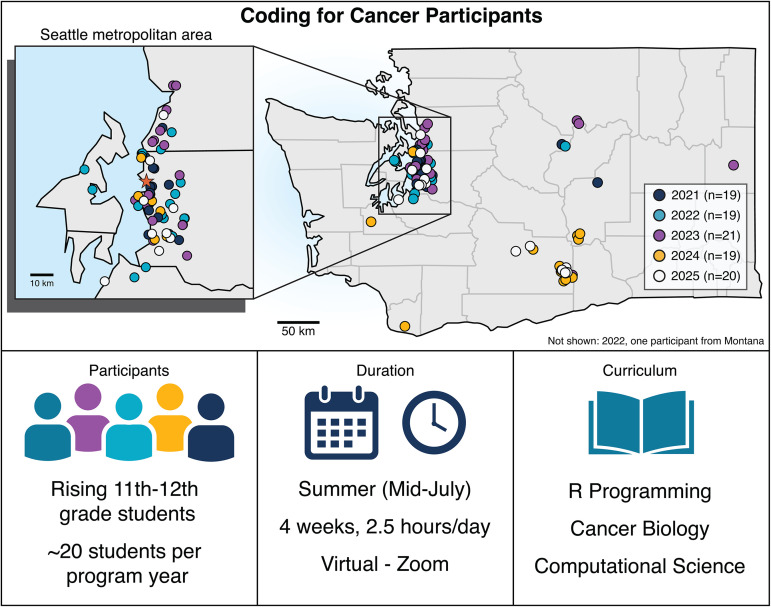
Overview of the Coding for Cancer program. Coding for Cancer is a 4-week virtual summer program for high school students to gain experience in computational biology and cancer research. Since its inception, Coding for Cancer has served over 90 students in the Pacific Northwest. The star indicates the location of the Fred Hutch Cancer Center Seattle Campus.

The Coding for Cancer Program at the Fred Hutch Cancer Center (Fred Hutch) is a virtual summer program that provides 20 11th and 12th-grade high school students with introductory experiences in computational biology and cancer research. This 4-week awareness-building program was initiated in 2021 in response to the COVID-19 pandemic as an alternative, fully virtual approach to in-person scientific education and outreach. As lockdown protocols were lifted, we maintained this platform for students geographically far from the Fred Hutch (and the greater Seattle area) as an opportunity to provide this educational experience without the commitments of commuting and lodging (Fig 1).

**Fig 2:**
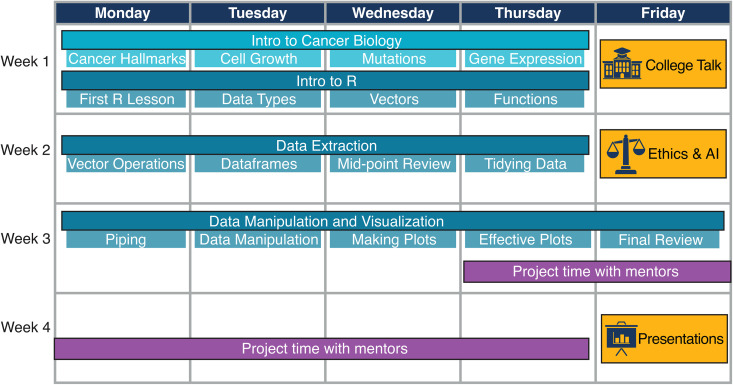
Coding for Cancer 4-week curriculum. The first week focuses on an introduction to cancer biology and R. The second and third weeks detail data processing and visualization workflows. The program culminates in a week-long research project in which students work together with mentor scientists. Additionally, professional development discussions are integrated into the program. Professional development topics included college admissions and bioethics.

As an introductory and exploratory program, Coding for Cancer aims to increase participant awareness in the computational biology and cancer research fields. The first three weeks of the curriculum focus on computational thinking, the R coding language, basic cancer biology, and professional development (Fig 2). In the final week, students work on independent research projects using data from the Cancer Genome Atlas with mentor scientists from the Fred Hutch and the University of Washington (UW).

As interest in data science, computer science, and computational biology rises among students, it is pivotal to empower the next generation of diverse thinkers and scientists to advance our knowledge in the sciences [1–3]. Learning computational biology may be intimidating or inaccessible, especially for students who have not yet had access to introductory learning experiences. Traditional teaching strategies on these topics are not widely available to some students, further perpetuating obstacles to diversifying the field [4].

In writing this Ten Simple Rules article, we aim to describe the components of our virtual education program, Coding for Cancer, that enable scientists and educators to foster youth interest in computational biology. We hope these rules, in addition to previously discussed rules focused on research experiences for high school students [5–7] and virtual learning [8,9], will provide a robust platform in which other education programs within and beyond the computational biology community can be modeled.

## Rule 1: Set students up for success in a virtual learning environment

Coding for Cancer is designed for students to learn about computational biology and gain awareness of potential careers within the field. While the program is open to all students, it specifically aims to provide access and opportunities to students furthest from these resources. This necessitates the program to be flexible in the support we provide to our participants. We offer financial support in the form of a participant award upon completion of the program to offset any loss of wages that might otherwise be earned through a summer job. We also provide material support through loaning laptops, monitors, and mobile hotspots for those who may face these obstacles as barriers to joining the program. This ensures that anyone, regardless of the setup in their home, can join and learn with us. Material requests are received by the instructional staff upon program acceptance and are mailed to students one month before the program begins, allowing time to test equipment at home. Most equipment issues, typically related to account logins, are resolved via email. Whenever additional assistance is needed, our IT staff provides support through email or remote login. By reducing any anxiety or obstacles linked to accessing resources and equipment, we are physically and emotionally supporting our students on their learning journey [10]. This allows us to immediately begin building positive relationships with our students and lay the foundation of our learning community.

In addition to hardware support, we offer various curricular supports to ensure that students interact with the materials in many different ways to fully participate and engage with the curriculum. We supply “Coding Cheat Sheets” that reference function usage and important syntax. Course materials such as slide decks and solutions for activities are readily accessible for students on Google Classroom. As summers are typically busy for students, especially those who may pursue school-related commitments or employment opportunities, we do not expect students to work on class material outside the scheduled program times. As such, we upload recordings of each session daily and host office hours outside of the program time so that students may access these resources when needed.

Two co-instructors and one program manager attend each live session. Typically, one co-instructor will lead the course material, while the other co-instructor provides technical support such as assisting individuals and answering questions in the chat. The program manager provides logistical support such as recording attendance, managing the Zoom call, and maintaining the daily schedule. These roles alternate between the instruction team on a daily basis and ensure that both the staff and students are supported for a seamless and engaging virtual learning experience.

## Rule 2: The (virtual) world is your oyster: Use a variety of web-based tools

**Fig 3:**
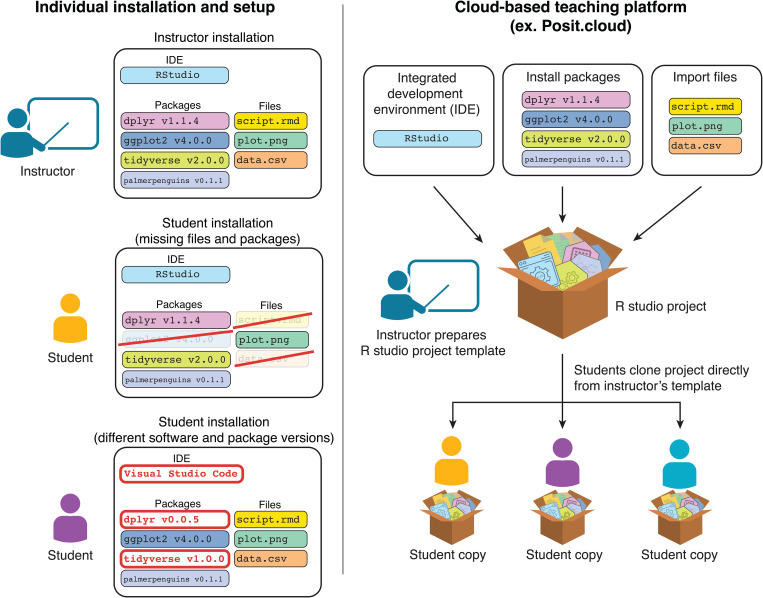
Cloud-based computing platforms streamline the logistics of software installation. Compared to individual installations where file versions may differ and installations may be incomplete, the use of cloud-based platforms such as Posit.cloud ensures the same computing environment for all members of the program. Software and packages are installed in a project template that the instructors create and distributed to students. Additionally, instructors have access to students’ workspaces to further troubleshoot issues.

There are many tools and programs available to foster a virtual learning environment; here, we describe some that were most useful for us. Web-based learning management systems such as Google Classroom enable students to access slide decks, worksheet keys, and video recordings. Cloud-based services such as Google Colab and Posit.cloud provide accessible computing platforms without the need for local installation on individual computers. As our program primarily focused on introducing R, we utilized Posit.cloud and RStudio, an integrated development environment. For each unit of the course, we first prepare a RStudio project by installing the necessary packages and uploading datasets and files. This project can then be used as a template that generates an identical, ready-to-go RStudio project for students (Fig 3). A messaging application such as Slack facilitates communication between students, instructors, and mentors during and outside of class hours. Finally, numerous open-source packages, such as learnr [11] and swirl (https://swirlstats.com), enable instructors to curate a virtual learning experience for their audience.

## Rule 3: Break the (virtual) ice: Establish an inclusive online culture

The online environment offers many advantages, from easing the burden of transportation and accessibility for participants to opening doors to different types of participation. However, it adds unique challenges in forming a community and a sense of belonging. To mitigate these impacts, we implemented various strategies to encourage an inclusive online culture in our daily practice and program design.

We begin each day with a Daily Warm Up activity, an ice-breaker style question or a short discussion, to check in with students and get to know one another. These prompts are typically not related to program content and are designed to invite varied aspects of a student’s identity into the classroom. Examples our students enjoyed included a Classroom Map that visualized where each student was calling in to the session from, discussing what kind of vegetable they would be, and sharing a country they would like to visit. Furthermore, we adopted various polling strategies aimed at easing the burden of participation, such as emoji reactions on Zoom, real-time poll response tools such as Mentimeter and Kahoot, and chat waterfalls where students submit their responses in the chat simultaneously. These simple questions can provide an opportunity to bring playfulness into our learning, while also creating a foundation for learning partnerships and communities [10].

A key Daily Warm-Up activity we implemented was Identity Artifacts, a visual representation of one’s many identities (avowed and ascribed) to be presented on an individual slide [12]. An example of an Identity Artifact, along with a list of Daily Warm-Up prompts is accessible on the Coding for Cancer GitHub repository. The Identity Artifact allowed instructors and students to bring non-academic aspects of their lives and identities into the online classroom, potentially finding commonalities, and advocate for a welcoming and respectful environment. Through this activity, students bonded over shared heritage, cultural backgrounds, and hobbies. By sharing important identities at the start of our program, we worked to center them as strengths, whereas traditional classrooms and scientific communities may diminish their importance [13].

## Rule 4: Model your learning. Teach by example

Much of learning and thinking in programming and coding is performed quietly and often unseen. How one develops the conceptual understanding and approach to tackling a coding question is not often explicitly taught and varies among instructor preferences. While some differences in approach may be purely stylistic, such as variable names or code formatting, others are more consequential, such as function choice and usage.

As instructors, we use ourselves to model our own learning. Similar to a live demonstration for a cooking class, we clarify our thought process in a visible manner by discussing our approach step-by-step and providing the rationale for our strategy. As an example of modeling the learning process, we describe a common scenario of encountering an error message and debugging. As a class, we voice our process by parsing the error message, locating where the error occurred, and finding potential solutions. We often show the process of searching through the documentation for definitions and syntax, or searching through public forums for alternative approaches. This simple act of demonstrating and explaining our process, rather than presenting the “right answer,” helps to demystify the coding process, and in this particular case, normalizes making mistakes when writing code.

## Rule 5: Encourage group work and collaborative work

Collaborative sensemaking enables students to engage with the curriculum while learning together and individually developing their understanding. To facilitate collaborative group work in a fully virtual program, we utilized Zoom breakout rooms in combination with Google Slides as a workspace for students to interact simultaneously and in real-time. Furthermore, as a technical note for instructors, Google Slides enables instructors to observe student progress and adjust timing or provide intermediate feedback much like one would in person. Groups were assigned for short group activities (~15 min) during lessons, and student groupings were rearranged every week so students had the opportunity to collaborate and further build rapport with their peers. When working on coding worksheets or assignments, we opened various breakout rooms to accommodate students’ preferences, depending on whether they want to work independently, in small groups, or collectively as a class. We found that it was important to provide a range of strategies for the students to engage with the material to deepen their understanding.

## Rule 6: Have fun exploring data

Dataframes are essential for organizing data into two-dimensional structures. However, students, especially first-time coders, may find navigating large dataframes intimidating due to the abundance of information presented at once. To encourage data exploration, we navigated datasets based on student interests such as popular culture, video games, and sports, before proceeding to more complex datasets. These datasets provided tangible and familiar data points for students and reduced the barriers to navigating dataframes. We particularly appreciated incorporating the palmerpenguins dataset in our data manipulation lessons as it provided features that were easily conceptualized, such as penguin body mass, bill depth, and flipper length [14]. Additionally, we created a makeshift dataset containing the cost, yield, and selling prices of crops from the Stardew Valley video game, and it was highly popular among students. In this manner, students practice data manipulation without the additional navigation of biological concepts and data that they might not be familiar with. Overall, we find that using tangible datasets when introducing data exploration and manipulation methods not only provides an additional level of engagement but also better prepares students to explore larger and more complex datasets.

## Rule 7: Support students’ professional development and career exploration journey

An educational program should not be limited to only presenting technical knowledge, but should also explore professional development opportunities to provide students with a view of potential career paths. In addition to introducing coding and computational biology, Coding for Cancer provides a platform for students to discuss their professional goals and seek future opportunities in computational biology and biomedical research. We dedicate at least one lesson a week, typically on Fridays, to professional development topics which include résumé building, career panels, college admissions, data literacy, and ethics. Students are given opportunities to ask questions and receive expertise from a scientific lens, which is beneficial for those who are looking to obtain further education in STEM, particularly for those who may not have access to other opportunities to learn these skills.

In today’s data-driven world, showcasing technical proficiency is more important than ever—and having a tangible credential to reflect the hard work and dedication students put into learning R coding further adds to their professional development. Upon completion of the program, students are issued a “Coding for Cancer: Introductory R” Credly accreditation badge that they may display on their LinkedIn profiles, CVs, and résumés to demonstrate their R coding competency. This recognition not only validates students’ skills but also empowers them to confidently showcase their expertise in professional settings.

Learning should not end when the program ends. Connect students to new opportunities, both local and abroad, that allow them to continue developing their skills. Upon completion of Coding for Cancer, students can apply for additional programs at the Fred Hutch Cancer Center and University of Washington, ranging from computational biology research to hands-on experimental research in the lab. In our 5-year history, we had 14 students participate in the Fred Hutch Explorers Virtual Internship, an academic year-long internship that pairs students with researchers to conduct biomedical research [15]. Four students took part in the Summer High School Internship Program (SHIP) for a summer research experience and three students in the SeattleSTATGrows Program, a biostatistics-focused summer research experience. These programs and internships provide students a wealth of opportunities to further develop their skills and advance their careers in meaningful ways.

## Rule 8: Learning from mentors: Work with mentor scientists on a final project

At the end of the program, students showcase their newfound programming skills through an independent research project of their choosing. Given our emphasis on cancer biology, we curated projects that highlighted real-world applications of the field such as investigating tumor suppressor genes and patient survival outcomes for specific cancer mutations. While we, the instructors, provide guidelines on expected deliverables, the project allows students to design their own hypothesis-driven research question and develop a methodology to answer it. As our program is designed for awareness building and exploration, this research project serves as a summative assessment for students’ learning. Additionally, the project provides students with a sense of ownership and autonomy in their learning and further facilitates their independent and critical thinking skills. This form of scientific discovery allows students to apply their learning and work collaboratively to refine their understanding [16,17].

**Fig 4:**
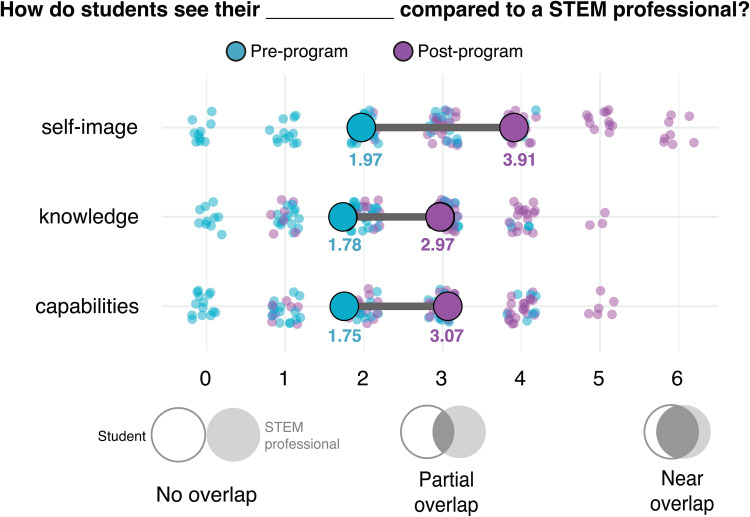
Coding for Cancer fosters students’ scientific identity. Student survey responses when asked how their self-image, knowledge, and capabilities compared to those of a STEM profession pre- and post-program. Mean response is indicated in colored circles. Responses were collected from program years 2021 (*n* = 14), 2022 (*n* = 19), 2024 (*n* = 17), and 2025 (*n* = 19). The 2023 cohort utilized a different survey that omitted this question, no data were collected.

Students are paired with scientific mentors, graduate students, postdocs, and staff scientists from Fred Hutch and the University of Washington communities, who help guide their ideas and provide feedback throughout their project. Before the start of the project, we provide mentors with instructional and technical support to engage and guide students on their projects. These scientific mentors serve not only as advisors for the projects but also as people whom the students can connect with for further career exploration. The mentored research project not only builds professional and technical skills but also serves as a platform to foster personal skill development, such as resilience, resourcefulness, and initiative. For many students, this research project is their first experience in conducting scientific research and interacting with science professionals, and, as such, these experiences together can strengthen students’ identities and their self-image as a STEM professional (Fig 4) [18,19].

## Rule 9: Harness various feedback strategies

The virtual nature of the program, in addition to the target audience of high school students, heightens the need for diverse yet structured feedback strategies. As such, we implement various models of feedback to gauge student understanding that are used to adjust lesson progression and daily pacing [20].

### Informal feedback

Throughout the program, we collect a variety of informal feedback as ways to measure student understanding and modify our teaching strategies [10]. We routinely used emoji reactions in the Zoom chat to depict comfort with a topic or skill. This routine not only gave the instructors valuable feedback in the moment to adjust pacing, but also served as a way for students to communicate with the teaching staff their understanding and familiarity with the topics covered. Additionally, at the end of each class period, we hold virtual open office hours, typically one hour after each class session, for students who prefer personalized discussion and assistance.

At the end of each week, the week’s topics are summarized in a weekly review to ensure that students understand the material and help gauge student learning progression. This also allows them the weekend to process and practice anything that they were still struggling with before moving on to the following week’s content.

### Formative feedback

At the end of each day, students are asked to complete an exit ticket survey- an anonymous, ungraded questionnaire that asks basic conceptual questions and invites feedback on the pacing and content of the lesson. Instructors review responses after every session and address any concerns or provides additional clarification the following day.

## Rule 10. Be flexible: Don’t be confined by a set curriculum

Computational and data science are rapidly evolving fields, and the program curriculum should reflect this dynamic nature. While the curriculum goals may be established before the start of each program, or adapted from previous years, it is important to build in flexibility to shift the curriculum as needed based on feedback, student background, and emerging topics.

A notable curriculum adjustment in our 2024 program occurred after the lesson on dataframe manipulation. Based on exit ticket responses, we observed that students were struggling with parsing and indexing dataframes. While the next lesson was planned to introduce the tidyverse modules, in response to this feedback, we instead shifted to an impromptu review on topics covered thus far, reinforcing key concepts before starting the new unit.

Another significant enhancement was the addition of the Intro to Cancer Biology modules during the first week. This section focuses on the fundamentals of cancer biology while emphasizing big data concepts relevant to biological and clinical data, which would be explored later. By providing context for the data early on, we aimed to establish a shared foundation and deepen students’ understanding before tackling more complex datasets.

The rise of AI tools also prompted a curriculum shift. Pre-program surveys revealed that students are either familiar with or have used AI tools in their daily lives. As such, we added a module focused on the ethics of AI, with the goal of encouraging discussion and bringing awareness to responsible AI use. Post-program surveys confirmed the impact of this addition, with many students highlighting the ethics session as one of the most engaging and insightful parts of the program.

## Conclusion

As interest in computational sciences increases among students, it is important to engage all students in a productive and relatable manner. Here, we described rules that enhance the virtual learning experience for both instructors and students. We advocate for tailoring a program that fosters collaborative and immersive learning despite being geographically dispersed and online. As such, these rules apply to any virtual learning platform within and beyond the computational and life sciences.
